# Whole-genome sequencing of *Leptospira interrogans* from southern Brazil: genetic features of a highly virulent strain

**DOI:** 10.1590/0074-02760170130

**Published:** 2018-02

**Authors:** Sérgio Jorge, Frederico Schmitt Kremer, Natasha Rodrigues de Oliveira, Gabrielle de Oliveira Sanches Valerio Navarro, Amanda Munari Guimarães, Christian Domingues Sanchez, Rafael Danelon dos Santos Woloski, Karine Forster Ridieri, Vinícius Farias Campos, Luciano da Silva Pinto, Odir Antônio Dellagostin

**Affiliations:** Universidade Federal de Pelotas, Centro de Desenvolvimento Tecnológico, Núcleo de Biotecnologia, Pelotas, RS, Brasil

**Keywords:** leptospirosis, genomics, bioinformatics, next-generation sequencing, Leptospira interrogans, zoonosis, phylogenetic analysis, whole-genome sequencing

## Abstract

**BACKGROUND:**

Leptospirosis is the most widespread zoonotic disease. It is caused by infection with pathogenic *Leptospira* species, of which over 300 serovars have been described. The accurate identification of the causative *Leptospira* spp. is required to ascertain the pathogenic status of the local isolates.

**OBJECTIVES:**

This study aimed to obtain the complete genome sequence of a virulent *Leptospira interrogans* strain isolated from southern Brazil and to describe its genetic features.

**METHODS:**

The whole genome was sequenced by next-generation sequencing (Ion Torrent). The genome was assembled, scaffolded, annotated, and manually reviewed. Mutations were identified based on a variant calling analysis using the genome of *L. interrogans* strain Fiocruz L1-130 as a reference.

**FINDINGS:**

The entire genome had an average GC content of 35%. The variant calling analysis identified 119 single nucleotide polymorphisms (SNPs), from which 30 led to a missense mutation. The structural analyses identified potential evidence of genomic inversions, translocations, and deletions in both the chromosomes.

**MAIN CONCLUSIONS:**

The genome properties provide comprehensive information about the local isolates of *Leptospira* spp., and thereby, could facilitate the identification of new targets for the development of diagnostic kits and vaccines.

Leptospirosis, a zoonotic disease, is caused by infection with pathogenic bacteria belonging to the genus *Leptospira*, and occurs worldwide. Susceptible hosts can be infected by direct contact with the urine of infected animals or through indirect exposure to leptospires in contaminated soil or water (Adler & Moctezuma 2010). The genus *Leptospira* comprises of twenty-two different species isolated from distinct hosts and environments, including ten pathogenic species *(L. interrogans*, *L. kirschneri*, *L. borgpetersenii*, *L. santarosai*, *L. noguchii*, *L. weilii*, *L. alexanderi*, *L. kmetyi*, *L. alstonii*, *and L. mayottensis*); five intermediate species (*L. inadai*, *L. broomii, L. fainei, L. wolffii*, and *L. licerasiae*); and seven saprophytic species (*L. biflexa*, *L. wolbachii*, *L. meyeri*, *L. vanthielii*, *L. terpstrae*, *L. idonii*, and *L. yanagawae*) (Bourhy et al. 2014). Pathogenic and intermediate species have been isolated from humans and animals and may be the cause of various mild clinical manifestations, while saprophytic species, which are environmental bacteria, do not cause the disease in humans or animals (Adler & Moctezuma 2010).

The genome of *Leptospira* spp. consists of two circular chromosomes that have a cumulative length that ranges from 3.9 to 4.6 Mb, a variation larger than that observed in other spirochaetes. This variability in the genome length confers the bacteria with an ability to live within diverse environments and to adapt to a wide range of hosts (Picardeau et al. 2008). However, detailed knowledge and understanding about the molecular pathogenesis and virulence evolution of *Leptospira* spp. is still unavailable (Xu et al. 2016). Next-generation DNA sequencing technology has been widely employed to perform several comparative analyses of gene diversity between saprophytic and pathogenic leptospires (Picardeau et al. 2008) and between different species of pathogenic leptospires (Xu et al. 2016). Sequencing new *Leptospira* isolates from various global sources generates additional data that can facilitate a better understanding about this pathogen.

*L. interrogans* isolate Piscina has previously been obtained from an abandoned swimming pool in southern Brazil. Our group characterised this isolate and found that it was highly virulent in the hamster model, causing lesions that are typically associated with severe leptospirosis, with an LD_50_ of ~2 leptospires. This indicated that *L. interrogans* Piscina may pose a risk to public health in the region (Forster et al. 2013). Due to its high virulence, this isolate has also been used as a challenge strain in experiments aimed to evaluate the protection afforded by novel recombinant vaccine candidates against leptospirosis in vaccinology laboratories (Forster et al. 2015).

In the present study, we performed the whole-genome sequencing, assembly, and annotation of *L. interrogans* strain Piscina in combination with functional and structural analyses. These genome features provide a deeper understanding about the pathogenesis of Piscina and may facilitate future improvements in its diagnosis and prevention.

## MATERIALS AND METHODS

*Leptospira sp. isolation and genomic DNA extraction* - *Leptospira* sp. was isolated from a water sample collected from an abandoned swimming pool in southern Brazil that contained dead rats, as previously described (Forster et al. 2013). Ten millilitres culture grown for seven days in Ellinghausen-McCullough-Johnson-Harris (EMJH) medium was inactivated in a water bath at 56°C for 30 min and centrifuged at 13,000 × *g* for 5 min. DNA was extracted using Illustra Bacterium Genomic Prep Mini Spin kit following the manufacturer's instructions (GE Healthcare, São Paulo, SP, Brazil). The extracted DNA was analysed by agarose gel electrophoresis to evaluate its integrity and quality, and subsequently was stored at -20°C.

*Whole-genome sequencing* - Bacterial genome sequencing was performed using the Ion Torrent PGM (Life Technologies, Saint Aubin, France) and 100 ng DNA. The DNA library was constructed using enzymatic fragmentation and adaptor ligation with the Ion Xpress Plus fragment library kit (Life Technologies). Fragment size selection was performed using E-Gel® SizeSelect 2% (Invitrogen). After diluting the library at 100 pM, template preparation, emulsion polymerase chain reaction, and ion sphere particle (ISP) enrichment were performed using the Ion One Touch template kit (Life Technologies). The ISPs were loaded and sequenced on a 318 chip (Life Technologies).

*Genome assembly and annotation* - *De novo* genome assembly was generated by MIRA (http://www.chevreux.org), Newbler (http://www.454.com/), and SPAdes (Bankevich et al. 2012), and their results were merged by CISA (Lin & Liao 2013) to generate a consensus assembly. The scaffolds of each chromosome were separated using BLAST searches against the genome of *L. interrogans* strain Fiocruz L1-130 (GenBank: AE016823.1, AE016824.1) and ordered using CAR (Lu et al. 2014) based on this same reference genome. Assembly gaps were closed using GMCloser (Kosugi et al. 2015), FGAP (Piro et al. 2014), and manual curation with the CLC Genome Workbench (http://www.clcbio.com). Genome annotation was performed using Genix (Kremer et al. 2016a) and manually reviewed using Artemis (Rutherford et al. 2000).

*In silico multilocus sequence typing (MLST), variant calling analysis, and structural analysis* - An *in silico* MLST analysis was performed using BLASTn searches against a database of alleles of the seven housekeeping genes from the scheme described by Boonsilp et al. (2013). All allele and sequence type data were downloaded from the PubMLST repository (http://pubmlst.org/).

To identify base-scale mutations, such as single-nucleotide polymorphisms (SNPs), a variant calling analysis was performed. The filtered sequencing reads were aligned to the reference genome of *L. interrogans* strain Fiocruz L1-130 using Segemehl (http://www.bioinf.unileipzig.de/Software/segemehl/) and an initial identification of INDELs was performed using SAMtools and BCFtools (https://github.com/samtools), which were processed locally and re-aligned using GATk (https://software.broadinstitute.org/gatk/) to improve the reliability of the alignments in the read mapping file. Finally, a new variant calling was performed using BCFtools, which was based on the refined mapping. SnpEff was used to predict the effect of each mutation (Cingolani et al. 2012).

During the final assembly, the genome of *L. interrogans* strain Piscina was aligned to the genome of *L. interrogans* strain Fiocruz L1-130 using Artemis Comparison Tool (ACT) (Rutherford et al. 2000) to identify structural variations (e.g., translocations, large insertions/deletions, inversions). To understand the plasticity of the *L. interrogans* genome, it was also compared to three other strains of *L. interrogans* - RCA, Prea, and Capivara - from the same sequence type (ST:17), which were reported by our group in a previous study (Kremer et al. 2016b). These strains were obtained from different mammal maintenance hosts in Pelotas, southern Brazil. As these genome sequences were published as draft genomes, the same reference-guided contig-ordering process employed for Piscina was used to reconstruct their chromosome structure.

*Phylogenetic analysis* - Mugsy (Angiuoli & Salzberg 2011) was used to identify syntenic regions shared between the genome of strain Piscina; draft genomes of the *L. interrogans* strains RCA, Prea, Capivara, and Aceguá that we have previously described (Kremer et al. 2016b); and the finished genomes of *L. interrogans* (nearest phylogenetic relatives) (Supplementary data, Table I) already available at GenBank. The regions present in all genomes were selected and merged into a single alignment, which was processed by the tools “TreeConstruction.Distance-Calculator” and “TreeConstruction.DistanceTreeConstructor” from the Phylo module from the Biopython package (http://biopython.org/) to generate a neighbour-joining tree, which was plotted using the interactive tree of life server (http://itol.embl.de/).

## RESULTS

*Genome properties* - The whole-genome shotgun sequencing of Piscina resulted in 689,451 reads with mean Phred score (Q) > 20, which represented coverage of ~30× when an *L. interrogans* genome of ~4.60 Mb, such as that of the Fiocruz L1-130 strain, was considered. The final assembly of chromosome I (4.22 Mb) contained 35 gaps and 3,318 predicted genes, while chromosome II (0.36 Mb) contained 2 gaps and 285 predicted genes. The lack of coverage in some regions may be a consequence of the drawbacks associated with *de novo* genome assembly from short reads NGS data, the presence of repetitive DNA elements and transposases, and the existence of regions in the genome with large variations in the CG content that may be over- or under-fragmented during the library construction. The entire genome had an average GC content of 35%. An overview of the results of the genome annotation is displayed in Table I and a deeper description of riboswitch-regulated genes is showed in Table II.

**TABLE I:** Overview of the results from the genome annotation for the genome of *Leptospira interrogans* strain Piscina

Chromosome	Genes	CDSs^a^	tRNAs	rRNAs	Regulatory^b^	ncRNAs^c^
I	3318	3234	37	3	3	4
II	285	285	0	0	1	0

a coding DNA sequences;

b regulatory elements (e.g.: riboswitches);

c other non-coding RNA genes that are not tRNAs nor rRNAs (e.g.: tmRNAs, Rnase P).

**TABLE II:** Riboswitch-regulated genes identified in the genome of *Leptospira interrogans* strain Piscina

Locus_tag	Chr	Gene location	Riboswitch location	Gene product
A9P81_2540	I	2834517:2836628(-)	2836734:2836914(-)	TonB-dependent receptor
A9P81_2899	I	3203281:3204768(+)	3203061:3203159(+)	Phosphomethylpyrimidine synthase
A9P81_3614	I	4060940:4061521(+)	4060606:4060737(+)	Uncharacterised protein
A9P81_3931	II	161571:163268(-)	159140:159319(-)	Acyl-CoA dehydrogenase

Chr: chromosome.

*Identification of pathogenesis-related genes of the Piscina isolate* - The annotation results indicated the presence of genes associated with virulence, such as those encoding sphingomyelinases, leptospiral immunoglobulin-like (Lig) proteins and conserved surface-exposed proteins (LipL32, LipL41, LipL21, and Loa22) that are only found in pathogenic leptospires. The landscape of pathogenesis-related genesis is presented in Supplementary data, Table II.

*In silico MLST profiling* - The results of the *in silico* MLST analysis are presented in Table III. As demonstrated by the combination of alleles, the Piscina isolate belongs to the ST 17 of the MLST scheme 1, which comprises the serogroup Icterohaemorrhagiae and serovars Icterohaemorrhagiae and Copenhageni.

*Identification of SNPs* - The variant calling analysis identified 119 SNPs, of which 30 had Q ≥ 40 and led to a missense mutation in comparison to that of the *L. interrogans* Fiocruz L1-130 reference genome. As demonstrated by the variant calling analysis (Table IV), the most mutated loci were LIC_11095 and LIC_13379, which contained six and four missense SNPs, respectively.

**Fig. 1:**
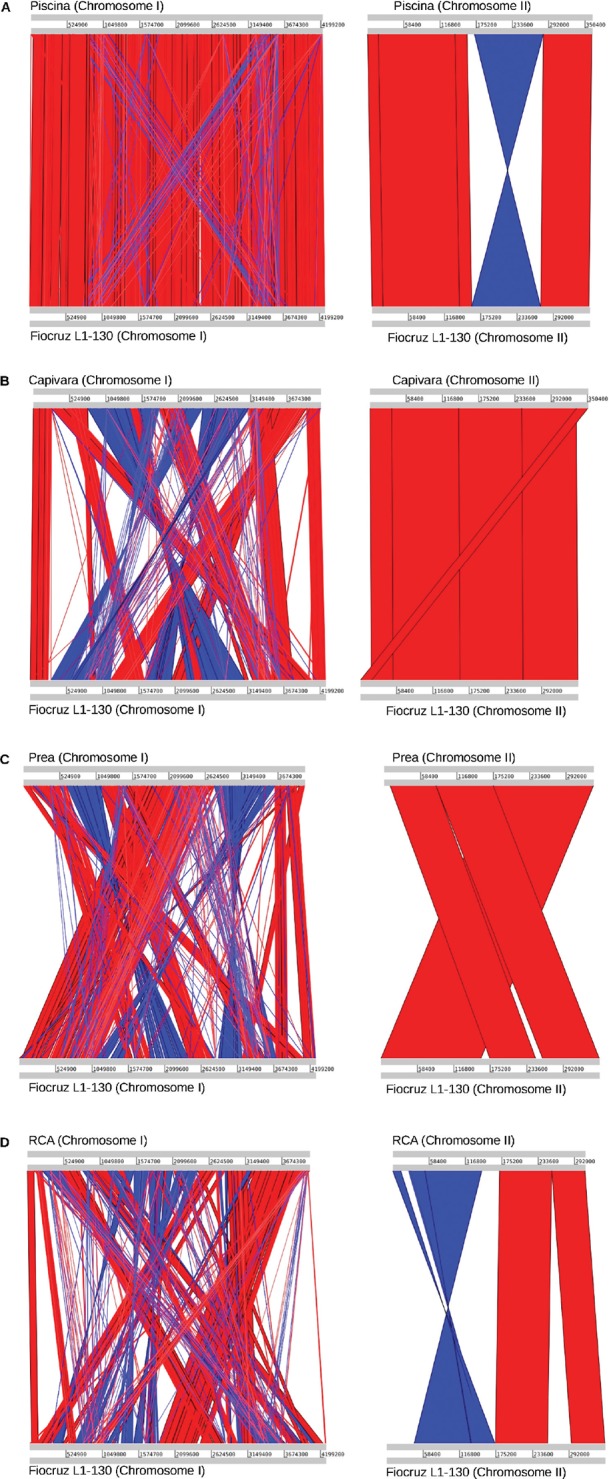
the genome structure reorganisation analysis generated by Artemis Comparison Tool (ACT), using the genome of *Leptospira interrogans* strain Fiocruz L1-130 as a reference, for the genome of the strains (A) Piscina, (B) Capivara, (C) Prea, and (D) RCA.

**Fig. 2:**
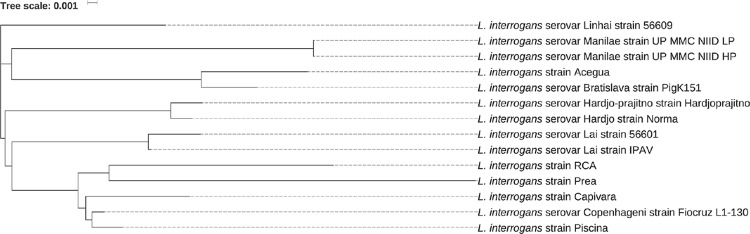
the findings from the neighbour-joining tree constructed based on the multiple alignment of syntenic regions reveal the relationship and genetic diversity among the Piscina strain, local isolates (RCA, Prea, Capivara, and Aceguá), and other whole-genome sequenced *Leptospira interrogans* strains.

*Comparative sequence analysis and phylogenetics* - The structural comparison generated by ACT based on the *L. interrogans* serovar strain Fiocruz L1-130 reference genome is presented in Fig. 1. All contigs were mapped to one of the two chromosomes, and no extrachromosomal element was identified (e.g., phage, plasmid). The structural analysis facilitated the identification of some potential events of genomic inversions, translocations, and deletions in both the chromosomes. The phylogenetic analysis performed based on alignment of the syntenic regions confirmed that *L. interrogans* serovar strain Fiocruz L1-130 and Piscina isolate belong to the same pathogenic group as portrayed in Fig. 2.

## DISCUSSION

The virulence of *Leptospira* spp. is known to be strain dependent (Adler & Moctezuma 2010), as highlighted by the sequencing of two *L. borgpetersenii* serovar Hardjo strains (Bulach et al. 2006). In a previous study, the high virulence of the Piscina isolate was confirmed in an animal model (Forster et al. 2013). Here, we generated the whole-genome sequence of Piscina isolate. The final assembly of the genome was 4.58 Mb, similar to that of the *L. interrogans* serovar Copenhageni Fiocruz L1-130 reference strain (Nascimento et al. 2004).

Along with the protein-coding genes, tRNAs, and rRNAs, we were able to identify other non-coding features, such as transfer-messenger RNAs (tmRNAs), riboswitches, RNase P, and clusters of regularly interspaced short palindromic repeat (CRISPR) loci (Table I). The majority of previous efforts to understand the pathogenesis were focused on protein-coding genes to identify virulence factors and new targets for vaccines and diagnosis; therefore, the annotation of non-coding features in *Leptospira* genomes was usually neglected. However, a previous study demonstrated that some of these non-coding features, such as riboswitches and CRISPR loci, are differentially distributed among saprophytic and pathogenic strains (Fouts et al. 2016).

Riboswitches are non-coding RNA motifs that are present in the 5′ untranslated regions (UTR) of some mRNAs and act as cis-regulatory elements that may suppress or activate gene expression. They are usually present in the genes associated with the synthesis of some vitamins (e.g., cobalamin, thiamine). Previous research has suggested that pathogenic species of *Leptospira* present complete riboswitch-regulated operons for the biosynthesis of cobalamin, indicating that they are able to respond to cobalamin levels and produce this metabolite from simpler molecules; however, these operons and pathways are incomplete in saprophytic species (e.g., *L. biflexa*) (Ricaldi et al. 2012). As indicated in Tables I-II, our analysis identified four riboswitch loci, which are located in the upstream region of the genes A9P81_2540, A9P81_2899, A9P81_3614, and A9P81_3931. Interestingly, the gene A9P81_2540, homologous to the gene LIC_12374 from *L. interrogans* strain Fiocruz L1-130, encodes a TonB-like outer-membrane protein that is responsible for cobalamin transport, and has been identified by our group as a potential vaccine target as it presents a large number of computationally-predicted surface-exposed epitopes (Grassmann et al. 2017).

Similar to riboswitches, CRISPRs are also present in a large number in pathogenic species but less frequently in saprophytic species, indicating that these two groups use different mechanisms to evade being infected by phages and to prevent the transformation by foreign plasmids. The other identified families of non-coding features, including tmRNAs and RNase P, are mainly associated with the regulation of gene expression and are widely distributed among microbial genomes (Hayes & Keiler 2010).

To further characterise the Piscina isolate, identification of genes encoding several pathogen species-specific antigens was performed. As expected, the sequenced genome presented genes encoding *Leptospira* virulencerelated factors and important potential vaccine candidates (Supplementary data, Table II). Some of these were surface-exposed proteins that act at the interface between the bacteria and host, are conserved among distinct serovars, and have been considered as suitable cross-protective vaccine candidates. Among these proteins are the OMPs, OmpL1, Lipl41, and LipL32; the Lig family proteins (LigA, LigB, and LigC), which contain bacterial immunoglobulin-like domains; and the lipoprotein Loa22, which is necessary for the virulence of *L. interrogans* and is considered to represent a good candidate for vaccine development (Dellagostin et al. 2017). In addition, the presence of genes encoding the set of six surface proteins of the leptospiral endostatin-like protein (Len) family (LenABCDEF), which are able to bind to the complement regulator factor H and mammalian extracellular matrix (ECM) proteins, was confirmed (Stevenson et al. 2007).

Among the main virulence factors identified were the haemolytic sphingomyelinases (e.g., sph2), which may facilitate virulence during migration through host tissues and are missing in the non-pathogenic *L. biflexa* (Narayanavari et al. 2015). Additional important components of leptospiral virulence that were identified included fliM, a protein required for full and correct assembly of the flagella (Fontana et al. 2016), and fcpA, a flagellar protein essential for bacterial translational motility and invasion of the host cells (Wunder et al. 2016).

According to the combination of alleles, the Piscina isolate belongs to ST 17 of the MLST scheme 1, which comprises the serogroup Icterohaemorrhagiae and serovars Icterohaemorrhagiae and Copenhageni (Table III). These results are in agreement with the early characterisation performed by Forster et al. (2013), which used the variable number of tandem repeats (VNTR) and the Sanger sequencing of the *rpoB* locus.

In the variant calling analysis, the most mutated loci were LIC_11095 and LIC_13379, with six and four missense SNPs, respectively. LIC_11095, which encodes an adenylate/guanylate cyclase (AGC), shows presence of orthologous genes found only in pathogenic and intermediary strains of *Leptospira* (Lehmann et al. 2013). They are described to be able to modulate the host's response to other pathogens, such as *Mycobacterium tuberculosis*, *Bordetella pertussis*, and *Pseudomonas aeruginosa* (Ahuja et al. 2004). LIC_13379 is annotated as a protease that contains the CAAX motif; however, its role in *Leptospira* virulence is poorly understood. By using the variant calling pipeline, we were able to identify 119 SNPs in the Piscina isolate, which are relatively low in number when compared to those of other *L. interrogans* strains (Xu et al. 2016). One explanation is that Ion Torrent platform has a high error-rate in homopolymeric regions, and some regions of low quality may contain other short variants, such as INDELs (insertions and deletions), that have not been identified. However, it may also indicate that Piscina and L1-130 are closely related strains, and may be a part of not only the same ST and serogroup, but also the same clonal group, which is also suggested from the results of structural genomics comparison (Fig. 1) and the phylogeny analysis (Fig. 2).

The structural comparison of the strains Piscina, RCA, Prea, and Capivara with the reference genome of the strain Fiocruz L1-130 allowed the identification of genomic inversions and/or translocations in all the genomes. Piscina showed the higher degree of structural similarity, although an inversion was observed in chromosome II, while Prea, RCA, and Capivara showed a large number of translocations and inversions in both the chromosomes, indicating that *L. interrogans* might present high plasticity in the genome organisation even when comparing strains from the same ST. Additionally, structural rearrangements have already been associated with a wide variety of phenotype-regulation processes; therefore, it is possible that their identification and characterisation may enhance understanding of some biological processes, such as adaptation to the host and immune response evasion. As a typical *L. interrogans* genome usually presents ~80 genes encoding transposases (e.g., strain Fiocruz L1-130), the identification of these rearrangement events in newly sequenced genomes is not unexpected. However, in contrast to the current research, they are usually only described when comparing non-closely related strains, such as those from different serogroups or serovars (Nascimento et al. 2004). While strain Piscina was obtained from a contaminated pool containing dead rats (environmental sample), the strains Prea, RCA, and Capivara were obtained from different mammal hosts (*Cavia aperea*, *Canis lupus familiaris*, and *Hydrochoerus hydrochaeris*, respectively). Therefore, it may suggest a potential relationship between host-adaptation and genome rearrangements, although our comparison is still limited by the small number of strains.

The availability of the whole genome sequence of a highly virulent *L. interrogans* strain isolated from southern Brazil provides researchers with a valuable opportunity to study differences in genome structures and facilitates the development of a comprehensive understanding of local isolates. The genome features of the local isolates contribute to comparative analyses with other sequenced genomes of the pathogenic *Leptospira* spp., thereby allowing the identification of new targets for the development of diagnostic kits and vaccines. Therefore, sequencing new local isolates provides genetic and epidemiological information that can improve existing knowledge of the *L. interrogans* infection.

*Nucleotide sequence accession numbers* - The whole genome shotgun projects have been deposited at DDBJ/EMBL/GenBank under the accessions CP018146.1 (Chromosome I) and CP018147.1 (Chromosome II).
