# Supplementary material for: Whole-genome sequencing of Leptospira interrogans from southern Brazil: genetic features of a highly virulent strain
Source: Mem Inst Oswaldo Cruz. 2018 Feb;113(2):80–6. doi: 10.1590/0074-02760170130 (PMC5722262; doi:10.1590/0074-02760170130)
Supplement: Supplementary file 1 [file 0074-0276-mioc-113-02-0080-Suppl01.pdf]

TABLE I

*Leptospira interrogans* serovars used in phylogenetic tree

| Specie | Serovar | Strain | GenBank | Status |
|---|---|---|---|---|
| *L. interrogans* | Bratislava | PigK151 | CP011410.1, CP011411.1 | Finished |
| *L. interrogans* | Copenhageni/Icterohaemorrhagiae | Piscina | CP018147.1, CP018146.1 | Finished |
| *L. interrogans* | Copenhageni | Fiocruz L1-130 | AE016823.1, AE016824.1 | Finished |
| *L. interrogans* | Hardjo-prajitno | Hardjoprajitno | CP013148.1, CP013147.1 | Finished |
| *L. interrogans* | Hardjo | Norma | CP012604.1, CP012603.1 | Finished |
| *L. interrogans* | Lai | 56601 | AE010300.2, AE010301.2 | Finished |
| *L. interrogans* | Lai | IPAV | CP001221.1, CP001222.1 | Finished |
| *L. interrogans* | Linhai | 56609 | CP006723.1, CP006724.1 | Finished |
| *L. interrogans* | Manilae | UP-MMC-NIID HP | CP011934.1, CP011935.1 | Finished |
| *L. interrogans* | Manilae | UP-MMC-NIID LP | CP011931.1, CP011932.1 | Finished |
| *L. interrogans* | Copenhageni/Icterohaemorrhagiae | Capivara | LJBQ01000000 | Draft |
| *L. interrogans* | Copenhageni/Icterohaemorrhagiae | Prea | LJBO01000000 | Draft |
| *L. interrogans* | Copenhageni/Icterohaemorrhagiae | RCA | LJBP01000000 | Draft |
| *L. interrogans* | Muenchen | acegua | LCZF01000000 | Draft |

TABLE II

List of pathogenesis-related genes and vaccine targets identified in the genome of *Leptospira interrogans* strain Piscina

| | Locus tag | | | |
|---|---|---|---|---|
| Gene | Piscina | Fiocruz L1-130 | Function | Reference |
| *lenA* | A9P81_3133 | LIC_12906 | Binding to laminin | (Stevenson et al. 2007) |
| *lenB* | A9P81_1050 | LIC_10997 | Binding to fibronectin and laminin | (Stevenson et al. 2007) |
| *lenC* | A9P81_3236 | LIC_13006 | Binding to fibronectin and laminin | (Stevenson et al. 2007) |
| *lenD* | A9P81_2481 | LIC_12315 | Binding to fibronectin and laminin | (Stevenson et al. 2007) |
| *lenE* | A9P81_3741 | LIC_13467 | Binding to fibronectin and laminin | (Stevenson et al. 2007) |
| *lenF* | A9P81_3490 | LIC_13248 | Binding to fibronectin and laminin | (Stevenson et al. 2007) |
| *lipL32* | A9P81_1441 | LIC_11352 | Binding to fibronectin, laminin and collagen type I, V, IV and XX | (Haake et al. 2000, Chaemchuen et al. 2011) |
| *ligA* | A9P81_0501 | LIC_10465 | Binding to fibronectin, laminin, tropoelastin and collagen type I and IV | (Lin et al. 2009) |
| *ligB* | A9P81_0500 | LIC_10464 | Binding to fibronectin, laminin, tropoelastin and collagen type I, III and IV | (Lin et al. 2009) |
| *lsa66* | A9P81_0300 | LIC_10258 | Binding to fibronectin and laminin | (Oliveira et al. 2011) |
| *lsa27* | A9P81_3121 | LIC_12895 | Binding to laminin | (Longhi et al. 2009) |
| *lsa20* | A9P81_1569 | LIC_11469 | Binding to laminin | (Mendes et al. 2011) |

↳

| Gene | Locus tag | | Function | Reference |
|---|---|---|---|---|
| | Piscina | Fiocruz L1-130 | | |
| *lsa25* | A9P81_2416 | LIC_12253 | Binding to laminin | (Domingos et al. 2012) |
| *lsa33* | A9P81_1981 | LIC_11834 | Binding to laminin | (Domingos et al. 2012) |
| *lic12976* | A9P81_3205 | LIC_12976 | Binding to laminin | (Lima et al. 2013) |
| *lp95* | A9P81_2909 | LIC_12690 | Binding to fibronectin and laminin | (Atzingen et al. 2009) |
| *lipl53* | A9P81_2247 | LIC_12099 | Binding to fibronectin, laminin and collagen type IV | (Oliveira et al. 2010) |
| *lsa21* | A9P81_0406 | LIC_10368 | Binding to fibronectin, laminin and collagen type IV | (Atzingen et al. 2008) |
| *lsa63* | A9P81_0352 | LIC_10314 | Binding to fibronectin, laminin and collagen type IV | (Vieira et al. 2010) |
| *lsa30* | A9P81_1152 | LIC_11087 | Binding to fibronectin and laminin | (Souza et al. 2012) |
| *ompl1* | A9P81_1027 | LIC_10973 | Binding to fibronectin and laminin | (Fernandes et al. 2012) |
| *ompl37* | A9P81_2425 | LIC_12263 | Binding to fibronectin and elastin | (Pinne et al. 2010) |
| *ompl47* | A9P81_3280 | LIC_13050 | Binding to fibronectin, laminin elastin and collagen type III | (Pinne et al. 2010) |
| *mfn1* | A9P81_1734 | LIC_11612 | Binding to fibronectin | (Pinne et al. 2012) |
| *mfn2* | A9P81_0773 | LIC_10714 | Binding to fibronectin | (Pinne et al. 2012) |
| *mfn6* | A9P81_1109 | LIC_11051 | Binding to fibronectin | (Pinne et al. 2012) |
| *mfn7* | A9P81_1538 | LIC_11436 | Binding to fibronectin | (Pinne et al. 2012) |
| *mfn9* | A9P81_0584 | LIC_10537 | Binding to fibronectin | (Pinne et al. 2012) |
| *tlyC* | A9P81_3374 | LIC_13143 | Binding to fibronectin, laminin and collagen IV | (Carvalho et al. 2009) |
| *Lipl21* | A9P81_0037 | LIC_10011 | Lipoprotein | (Cullen et al. 2003) |
| *lemA* | A9P81_1117 | LIC_11058 | Not expressed in strains used for vaccine production, but up-regulated in highly virulent strains | (Zeng et al. 2015) |
| *lipl45* | A9P81_1765 | LIC_11643 | Mutated in strain with medium virulence | (Zeng et al. 2015) |
| *lipl41* | A9P81_3196 | LIC_12966 | Lipoprotein | (Shang et al. 1996) |
| *FcpA* | A9P81_3397 | LIC_13166 | Flagelin | (Wunder et al. 2016) |
| *fliM* | A9P81_1983 | LIC_11836 | Required for the assembly of the flagellum. Mutation in this gene leads to attenuation of the strain | (Fontana et al. 2016) |
| *loa22* | A9P81_0234 | LIC_10191 | Virulence factor | (Ristow et al. 2007) |
| *lsa24* | A9P81_0406 | LIC_10368 | Binding to extracellular matrix. Only found in pathogenic strains | (Barbosa et al. 2006) |
| *lsa32* | A9P81_1154 | LIC_11089 | Binding to extracellular matrix | (Domingos et al. 2015) |
| *lp30 L* | A9P81_3106 | LIC_12880 | Binding to extracellular matrix | (Oliveira et al. 2011) |
| LIC_12238 | A9P81_2401* | LIC_12238 | binding to extracellular matrix | (Oliveira et al. 2011) |
| *clpA* | A9P81_2176 | LIC_12017 | Chaperone | (Zeng et al. 2015) |
| *sph2* | A9P81_2843 | LIC_12631 | Hemolytic activity | (Narayanavari et al. 2015) |

## REFERENCES


Atzingen MV, Barbosa AS, de Brito T, Vasconcellos SA, de Morais ZM, Lima DM, et al. Lsa21, a novel leptospiral protein binding adhesive matrix molecules and present during human infection. BMC Microbiol. 2008; 8: 70.

Atzingen MV, Gómez RM, Schattner M, Pretre G, Gonçales AP, de Morais ZM, et al. Lp95, a novel leptospiral protein that binds extracellular matrix components and activates e-selectin on endothelial cells. J Infect. 2009; 59(4): 264-76.

Barbosa AS, Abreu PAE, Neves FO, Atzingen MV, Watanabe MM, Vieira ML, et al. A newly identified leptospiral adhesin mediates attachment to laminin. Infect Immun. 2006; 74(11): 6356-64.

Carvalho E, Barbosa AS, Gómez RM, Cianciarullo AM, Hauk P, Abreu PA, et al. Leptospiral TlyC is an extracellular matrix-binding protein and does not present hemolysin activity. FEBS Lett. 2009; 583(8): 1381-5.

Chaemchuen S, Rungpragayphan S, Poovorawan Y, Patarakul K. Identification of candidate host proteins that interact with LipL32, the major outer membrane protein of pathogenic *Leptospira*, by random phage display peptide library. Vet Microbiol. 2011; 153(1-2): 178-85.

Cullen PA, Haake DA, Bulach DM, Zuerner RL, Adler B. LipL21 is a novel surface-exposed lipoprotein of pathogenic *Leptospira* species. Infect Immun. 2003; 71(5): 2414-21.

Domingos RF, Fernandes LG, Romero EC, de Morais ZM, Vasconcellos SA, Nascimento ALTO. Novel *Leptospira interrogans* protein Lsa32 is expressed during infection and binds laminin and plasminogen. Microbiology. 2015; 161(4): 851-64.

Domingos RF, Vieira ML, Romero EC, Gonçales A, de Morais ZM, Vasconcellos SA, et al. Features of two proteins of *Leptospira interrogans* with potential role in host-pathogen interactions. BMC Microbiol. 2012; 12: 50.

Fernandes LG V, Vieira ML, Kirchgatter K, Alves IJ, de Morais ZM, Vasconcellos SA, et al. Ompl1 is an extracellular matrix- and plasminogen-interacting protein of *Leptospira* spp. Infect Immun. 2012; 80(10): 3679-92.

Fontana C, Lambert A, Benaroudj N, Gasparini D, Gorgette O, Cachet N, et al. Analysis of a spontaneous non-motile and avirulent mutant shows that fliM is required for full endoflagella assembly in *Leptospira interrogans*. PLoS ONE. 2016; 11(4): e0152916.

Haake DA, Chao G, Zuerner RL, Barnett JK, Barnett D, Mazel M, et al. The leptospiral major outer membrane protein LipL32 is a lipoprotein expressed during mammalian Infection. Infect Immun. 2000; 68(4): 2276-85.

Lima SS, Ching ATC, Fávaro RD, da Silva JB, Oliveira MLS, Carvalho E, et al. Adhesin activity of *Leptospira interrogans* lipoprotein identified by *in vivo* and *in vitro* shotgun phage display. Biochem Biophys Res Commun. 2013; 431(2): 342-7.

Lin Y-P, Greenwood A, Yan W, Nicholson LK, Sharma Y, McDonough SP, et al. A novel fibronectin type III module binding motif identified on C-terminus of *Leptospira* immunoglobulin-like protein, LigB. Biochem Biophys Res Commun. 2009; 389(1): 57-62.

Longhi MT, Oliveira TR, Romero EC, Goncales AP, de Morais ZM, Vasconcellos SA, et al. A newly identified protein of *Leptospira interrogans* mediates binding to laminin. J Med Microbiol. 2009; 58: 1275-82.

Mendes RS, Von Atzingen M, de Morais ZM, Gonçales AP, Serrano SMT, Asega AF, et al. The novel leptospiral surface adhesin Lsa20 binds laminin and human plasminogen and is probably expressed during infection. Infect Immun. 2011; 79(11): 4657-67.

Narayanavari SA, Lourdault K, Sritharan M, Haake DA, Matsunaga J. Role of sph2 gene regulation in hemolytic and sphingomyelinase activities produced by *Leptospira interrogans*. PLoS Negl Trop Dis. 2015; 9(8): 1-23.

Oliveira R, de Morais ZM, Gonçales AP, Romero EC, Vasconcellos SA, Nascimento ALTO. Characterization of novel OmpA-like protein of *Leptospira interrogans* that binds extracellular matrix molecules and plasminogen. PLoS ONE. 2011; 6(7): e21962.

Oliveira TR, Longhi MT, Gonçales AP, de Morais ZM, Vasconcellos SA, Nascimento AL. LipL53, a temperature regulated protein from *Leptospira interrogans* that binds to extracellular matrix molecules. Microbes Infect. 2010; 12(3): 207-17.

Pinne M, Choy HA, Haake DA. The OmpL37 surface-exposed protein is expressed by pathogenic *Leptospira* during infection and binds skin and vascular elastin. PLoS Negl Trop Dis. 2010; 4(9): e815.

Pinne M, Matsunaga J, Haake DA. Leptospiral outer membrane protein microarray, a novel approach to identification of host ligand-binding proteins. J Bacteriol. 2012; 194(22): 6074-87.

Ristow P, Bourhy P, McBride FWDC, Figueira CP, Huerre M, Ave P, at al. The OmpA-like protein Loa22 is essential for leptospiral virulence. PLoS Pathog. 2007; 3(7): 894-903.

Shang ES, Summers TA, Haake DA. Molecular cloning and sequence analysis of the gene encoding LipL41, a surface-exposed lipoprotein of pathogenic *Leptospira* species. Infect Immun. 1996; 64(6): 2322-30.

Souza NM, Vieira ML, Alves IJ, de Morais ZM, Vasconcellos SA, Nascimento AL. Lsa30, a novel adhesin of *Leptospira interrogans* binds human plasminogen and the complement regulator C4bp. Microb Pathog. 2012; 53(3-4): 125-34.

Stevenson B, Choy HA, Pinne M, Rotondi ML, Miller MC, DeMoll E, et al. *Leptospira interrogans* endostatin-like outer membrane proteins bind host fibronectin, laminin and regulators of complement. PLoS ONE. 2007; 2(11): e1188.

Vieira ML, de Morais ZM, Gonçales AP, Romero EC, Vasconcellos SA, Nascimento AL. Lsa63, a newly identified surface protein of *Leptospira interrogans* binds laminin and collagen IV. J Infect. 2010; 60(1): 52-64.

Wunder EA, Figueira CP, Benaroudj N, Hu B, Tong BA, Trajtenberg F, et al. A novel flagellar sheath protein, FcpA, determines filament coiling, translational motility and virulence for the *Leptospira* spirochete. Mol Microbiol. 2016; 101(3): 457-70.

Zeng LB, Zhuang XR, Huang LL, Zhang YY, Chen CY, Dong K, et al. Comparative subproteome analysis of three representative *Leptospira interrogans* vaccine strains reveals cross-reactive antigens and novel virulence determinants. J Proteomics. 2015; 112: 27-37.
